# Detection and stage classification of Plasmodium falciparum from images of Giemsa stained thin blood films using random forest classifiers

**DOI:** 10.1186/s13000-020-01040-9

**Published:** 2020-10-23

**Authors:** Syed Saiden Abbas, Tjeerd M. H. Dijkstra

**Affiliations:** 1 Institute for Computing and Information Sciences, Radboud University Nijmegen, Nijmegen, Netherlands; 2 Department for Women’s Health, University Clinic Tübingen, Tübingen, Germany; 3 Max Planck Institute for Developmental Biology, Tübingen, Germany

## Abstract

**Background:**

The conventional method for the diagnosis of malaria parasites is the microscopic examination of stained blood films, which is time consuming and requires expertise. We introduce computer-based image segmentation and life stage classification with a random forest classifier. Segmentation and stage classification are performed on a large dataset of malaria parasites with ground truth labels provided by experts.

**Methods:**

We made use of Giemsa stained images obtained from the blood of 16 patients infected with Plasmodium falciparum. Experts labeled the parasite types from each of the images. We applied a two-step approach: image segmentation followed by life stage classification. In segmentation, we classified each pixel as a parasite or non-parasite pixel using a random forest classifier. Performance was evaluated with classification accuracy, Dice coefficient and free-response receiver operating characteristic (FROC) analysis. In life stage classification, we classified each of the segmented objects into one of 8 classes: 6 parasite life stages, early ring, late ring or early trophozoite, mid trophozoite, early schizont, late schizont or segmented, and two other classes, white blood cell or debris.

**Results:**

Our segmentation method gives an average cross-validated Dice coefficient of 0.82 which is a 13% improvement compared to the Otsu method. The Otsu method achieved a True Positive Fraction (TPF) of 0.925 at the expense of a False Positive Rate (FPR) of 2.45. At the same TPF of 0.925, our method achieved an FPR of 0.92, an improvement by more than a factor two. We find that inclusion of average intensity of the whole image as feature for the random forest considerably improves segmentation performance. We obtain an overall accuracy of 58.8*%* when classifying all life stages. Stages are mostly confused with their neighboring stages. When we reduce the life stages to ring, trophozoite and schizont only, we obtain an accuracy of 82.7*%*.

**Conclusion:**

Pixel classification gives better segmentation performance than the conventional Otsu method. Effects of staining and background variations can be reduced with the inclusion of average intensity features. The proposed method and data set can be used in the development of automatic tools for the detection and stage classification of malaria parasites. The data set is publicly available as a benchmark for future studies.

## Background

The conventional method of diagnosing malaria is the microscopic examination of blood films using Giemsa staining[1]. It is inexpensive and reliable but requires considerable expertise and training of health care workers [1]. It is considered the most efficient method for the study of parasites in different stages and the quantification of parasitemia [2].

A number of studies have been performed for the detection of parasites using digital images of Giemsa-stained blood films. Table 1 gives a description of the datasets used in previous studies. Most studies were performed on datasets with a small number of parasites except the study by Lindner et al. [3]. Table 2 gives an overview of the methods applied in previous studies. Most of the previous studies were limited to the detection of parasites except a few studies, which performed detection as well as stage classification. Previous studies applied methods based on histogram thresholding (color similarity, Otsu, local and adaptive thresholding etc.), and morphological operations as given in Table 2 and [4–12]. Most studies used only the green color channel for detection of parasites. There is not much literature on parasite detection using pixel classification except [13] and [14]. The study by Diaz et al. [13] presents pixel classification using different classifiers in different color spaces but this study was performed with only 60 parasites. The study by Vink et al. [14] was applied to fluorescent stained parasites. Table 2 shows that in previous studies stage classification was performed mainly with ring, trophozoite, schizont and gametocyte stages. A detailed literature review was recently published including other parasites than Plasmodium falciparum and other staining techniques than Giemsa [15].

**Table 1:** Data sets. RBCs = red blood cells, WBCs = white blood cells

Study	Number of images	RBCs	WBCs	Parasites	Cultured or patients	Data public
Linder [3]	549 per patient			8329	In vivo	Yes
Walliander [34]	473-505	32698	476		No information	No
Malihi [21]	363	238		125	No information	No
Savkare [22]	15				No information	No
Tek [31]	630	3431	151	669	No information	No
Diaz [28]	450	11844		713	In vitro	No
Diaz [13]	25	1226		60	No information	No
Ruberto [30]	12	1910	2	479	No information	No

**Table 2:** Analysis methods

Study	Color channel	Methods	Task
Linder [3]	Green	Histogram thresholding,	Detection of parasite regions
		support vector machine (SVM)	
Walliander [34]	Green	Adaptive histogram thresholding	Segmentation and counting of RBCs
Malihi [21]	Green	Extraction of cell mask with Otsu	Detection of parasites
		thresholding, K-nearest neighbour (k-NN)	
Savkare [22]	Green	Otsu thresholding, watershed segmentation, SVM	Segmentation of RBCs, detection of parasites
Tek [31]	Grey	Rao’s method, K-nearest neighbour	Detection of parasites, stage classification:
			ring, trophozoite, schizont, gametocyte
Diaz [28]	RGB	Color space classification, Inclusion-Tree	Detection of parasite, classification of stages:
		representation, SVM	ring, trophozoite, schizont
Diaz [13]	RGB, Normalized	Pixel classification, k-NN	Detection of parasite with pixel classification
	RGB HSV, YCbCr		
Ruberto [30]	Green, HSV, RGB	Morphological approach, color	Segmentation of RBCs, detection of parasites, stage
		histogram similarity	classifications: mature trophozoite, immature trophozoite,
			gametocyte

The contribution from this study is twofold. First, we perform image segmentation with pixel classification and evaluate it with cross-validated accuracy, Dice coefficient and free-response characteristic curves (FROC). Second, we perform a detailed analysis of the parasite life stage classification with early, late and mature stages of the parasite as opposed to previous studies with only ring, trophozoite and schizont stages. We also include white blood cells (WBCs) and debris in the classification task as this inclusion improved performance.

## Methods

### Data description

**Fig. 1:**
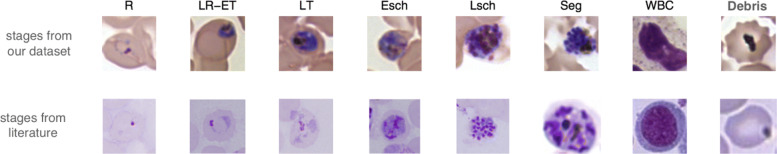
Stages of malaria parasite from our data set and literature [17]. See text for explanation of abbreviations

The images used in this study were collected from patients in Gambia, see Lemieux et al. [16] for details. Plasmodium falciparum parasites were ex-vivo cultured between 24 and 48 h. Typically, parasites and red blood cells look dark purple and light pink respectively in Giemsa stained images [13] as shown in Fig. 1 and Fig. S4 of Lemieux et al. [16]. Images vary from patient to patient due to staining variability and differences in the red blood cell density. Each image was a composite of 4 by 4 images of 1382 by 1030 pixels each, which we separated for analysis. In total, the data set comprised 837 images from 16 patients. We dropped one patient (coded 804 in [16]) as the staining was flawed in this patient.

### Ground truth image segmentation

We manually selected all dark-stained blobs which included parasites, white blood cells and debris. We cropped the images around each of the dark-stained blobs. We applied Otsu thresholding on the green channel for initial segmentation of parasites. We manually changed the threshold to segment each parasite (or white blood cell or debris) and adjusted the segmentation by hand, if needed. We filled and cleaned the segmented regions using morphological operations. In detail we used Matlab’s imfill() with a connectivity of 8 and Matlab’s imdilate() with a disc of radius 5 pixels as structuring element. We combined all segmented cropped images into a ground truth binary image.

### Labeling of parasite life stages

The cropped images were presented to malaria experts who classified parasites into ring (R), late ring or early trophozoite (LR-ET), mid trophozoite (MT), early schizont (Esch), late schizont (Lsch), segmenter (Seg), white blood cells (WBC) or debris. The parasite life stages were described as follows. R = light ring, LR/ET = fat rings with dark cytoplasm, very small trophozoites with pigment; rif stage, MT = pigment, vacuole, mid-size, LT = two nuclei, very large, Esch = more than two nuclei, dark staining, very large, Lsch = still has RBC, but clear merozoites, Seg = no RBC (or very faint outline); bunch of grapes. Debris is a left-over category for those blobs that could not be clearly classified as parasites or WBCs. Figure 1 shows images of parasites in different life stages from our dataset and from [17]. The size of parasites increases from early to late stages which can be observed from Fig. 1. There were a total of 2911 parasites in different life stages, see Table 3. As there is relatively limited class imbalance in the number of parasites in different life stages, we saw no need to adjust for class imbalance. The focus in this study is not on classification of WBC or debris, so number of cases in those categories matters less.

**Table 3:** Number of parasites in each stage

R	LR-ET	MT	LT	Esch	Lsch	Seg	WBC	Debris
538	307	332	367	714	446	207	50	683

To alleviate the burden we had several malaria experts classify all images, but each image was classified by a single expert. To benchmark the performance of our life stage classification we had a second malaria expert reclassify a balanced subset of 115 parasites into the same categories.

### Image features and pixel sampling

**Fig. 2:**
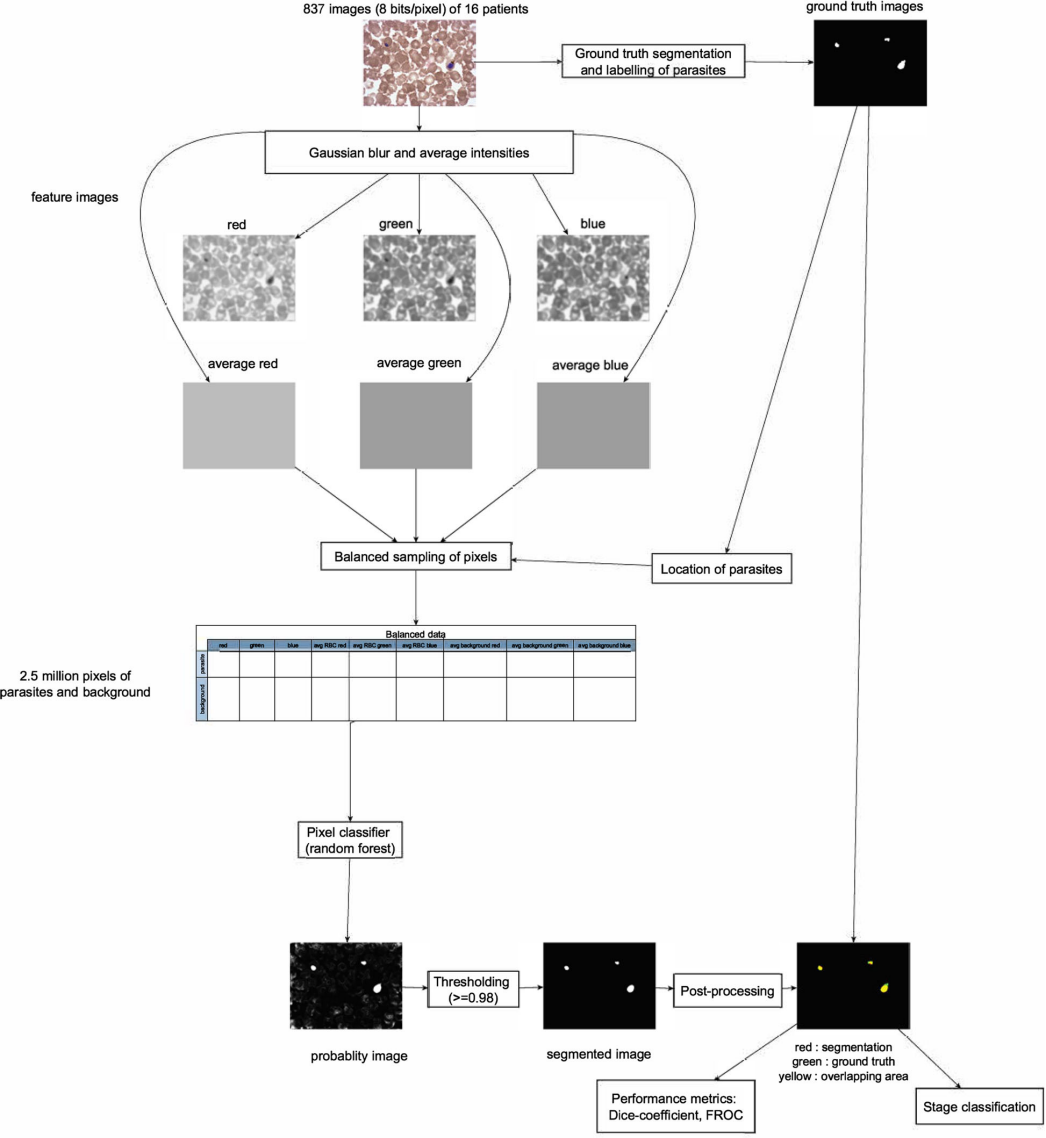
Flowchart of the parasite image segmentation method

Figure 2 gives an overview of the image processing pipeline. We smoothed each image by convolution with a Gaussian filter. The amount of smoothing is determined by the standard deviation of the Gaussian filter. A standard deviation of zero amounts to no smoothing, effectively keeping the original image, while a standard deviation much larger than the size of the image leads to an image where every pixel has the same value, the mean intensity of the original image. We varied the standard deviation of the Gaussian filter to find the optimal value for pixel classification. We ran two main analyses. In the first one, we performed pixel classification with three features, the pixel values of the blurred red, green and blue channels. In second analysis, we corrected for background and staining effects by including the average intensities of the whole image in all color channels in the pixel classification, thus increasing the number of features per pixel from 3 to 6. We also ran one control analysis where we investigated the use of two standard deviations of the gaussian blur but found no clear performance benefit over using a single one (results not shown). Two levels of gaussian blur could improve performance as one of these levels would serve for background correction and the second for smoothing. Results of this control analysis show that simply averaging each image is enough for background correction.

We considered two classes for pixel segmentation: parasite pixels and non-parasite pixels. Parasite pixels included pixels from parasites, white blood cells and debris while non-parasite pixels included pixels from background and red blood cells. As the parasites cover about 1% on average of each image, there is severe class imbalance. A trivial classifier that assigns each pixel to the non-parasite class would achieve a classification accuracy of 99%. To mitigate class imbalance, we used balanced sampling by choosing an equal number of parasite and non-parasite pixels. We randomly selected 2.5 million pixels of parasites and 2.5 million pixels of non-parasites for training. Using a larger number of pixels did not improve results. We ran a random forest classifier on the training data. The random forest classifier fits trees to randomly selected sub-sets of the pixel data. Each tree classifies a pixel as a parasite or a non-parasite. The probability (0≤*p*≤1) of a pixel to belong to a parasite is obtained by dividing the number of times a pixel is classified as parasite by the total number of trees. These pixel probabilities form so-called probability images and by thresholding them we obtained segmented images. We choose a threshold value of *θ*_*pm*_=0.98 based upon prior knowledge of the image area fraction covered by parasite pixels. We show in the Results section that this choice of the threshold is close to the optimal one. We performed post-processing on the segmented images with the morphological operation of filling, same parameters as for the ground truth images. Post-processing assures that segmented objects do not have holes due to misclassified pixels inside the segmented objects. We removed those segmented objects which had an area less than 100 pixels. This choice of 100 pixels is based on the area of the smallest parasite.

### Segmentation evaluation

We used three metrics to evaluate the performance of pixel classification: the training set accuracy, the Dice coefficient and the free-response receiver operating characteristic (FROC). For all metrics, we used leave-one-out cross-validation (LOOCV) to quantify predictive performance: we fitted the random forest to pixels from 15 patients and evaluated performance on the 16th patient whose images were not used in training. We averaged performance across the 16 left-out patients to obtain the metrics reported below.

**Pixel classification accuracy:** Pixel classification accuracy is defined as the number of correctly classified pixels divided by the total number of pixels. This metric evaluates performance on the balanced data set and hence baseline performance is 50%.

**Dice coefficient:** The Dice coefficient (*D*_*c*_) quantifies the overlap between segmented and ground truth foreground pixels. It is also known as the F1-measure. It is defined as:

$$D_{c} = \frac{2|I_{s} \cap I_{g}|}{(|I_{s}|+|I_{g}|)}, $$

where *I*_*s*_ and *I*_*g*_ are the segmented and ground truth images respectively and |*x*| denotes the number of foreground pixels in *x*. The value of the Dice coefficient varies between zero and one. It is zero when ground truth and segmented images have no overlap and it is one when they exactly coincide. As foreground pixels only cover around 1% of each image, baseline performance of a random classifier is also 1%. We summarized the Dice coefficient for all images from a patient by the median and over all patients by the mean.

**Free-response receiver operating characteristic:** The free-response receiver operating characteristic (FROC) is a generalization of the receiver operating characteristic (ROC) to the case where there are multiple possible false positives. This can occur in our setting as objects (blobs in an image) might be segmented that do not overlap with any of the ground truth objects (targets). To calculate the FROC one needs to specify when a parasite is detected. We considered any object in the segmented image as a detected and localized parasite, when it had a minimum Dice coefficient of 0.2 with a target parasite. This minimum Dice coefficient is called the acceptance Dice coefficient [18]. Any parasite localized and detected within the acceptance Dice coefficient is considered a true positive. All segmented objects that are not detected through the acceptance Dice coefficient, are considered false positives. All target parasites, which do not have any segmented parasite within the acceptance Dice coefficient are considered false negatives. The number of false negatives follows from the number of targets minus the number of true positives. Unlike ROC analysis there are no true negatives in FROC analysis.

In FROC analysis, we draw a curve for the true positive fraction (TPF) as a function of the false positive rate (FPR). The true positive fraction is defined as the number of true positives divided by total number of targets in all images. The false positive rate is defined as the number of false positives divided by the number of images. Values of the true positive fraction are between 0 and 1 while values of false positive rates (FPR) can be greater than 1, which is contrary to the conventional ROC analysis. In this study, we fixed the FPR between 0 and 1.5 for comparison purposes. True and false positives are obtained by changing the value of *θ*_*pm*_, the threshold of the probability map. We obtained 40 different thresholds by changing the probability threshold from 0.98 to 1. For every threshold, we obtained a segmented image on which we performed post processing as detailed in the segmentation section.

We used a random guessing model as a reference. In this model a random locations in the image are chosen as parasite locations. Zou et al. [18] use radius as acceptance criterion instead of the Dice coefficient as we do. As it is not clear how to specify a random guessing model based on the Dice coefficient, we picked a conservative acceptance radius of twice the radius of the largest parasite (2*78 = 156 pixels). The probability of a random location of a parasite falling within the acceptance radius is the ratio between the area of a circle with radius *r* and area *Σ* of the image:

$$p = \frac{\pi r^{2}}{\Sigma}. $$

The guessing model is defined as [18]:

$$TPF = 1-\exp(-FPR\times \phi), $$

where *ϕ* is defined as the odds $\frac {p}{1-p}$. We calculated the area under the curves of the guessing model and of the data. A higher difference of the area between the curves of the guessing model and the data means a better detection and localization of parasites. At the expense of greater complexity, FROC analysis provides insight into the trade-off between detecting a larger fraction of the true parasites and the false positives.

### Life stage classification

We considered intensity, shape, moment and Haralick features for life stage classification. Intensity features included mean, standard deviation, median and quartile intensity features. Shape features were area, perimeter, minimum radius, maximum radius, mean radius and standard deviation of radius. Moment features were major axis, orientation and eccentricity. Haralick features were texture features as reported in [19]. In total we calculated 112 features for each of the three color channels. All features were calculated using EBImage [20]. As we did for the evaluation of segmentation, we performed LOOCV and averaged accuracy across 16 patients.

We performed five analyses for a detailed study of the life stage classification performance. First, we performed stage classification separately with each channel to find out which of the channels is more explanatory for the stage classification. Second, we performed stage classification with all channels to explore the combined effect of all channels. Third, we combined the early and late sub-stages into one stage to perform stage classification with only three life stages: ring, trophozoite and schizont. Fourth, we evaluated stage classification by excluding white blood cells and debris. Fifth, we evaluated stage classification with 11 important features, which we obtained from feature importance with the random forest classifier.

### Otsu segmentation

Otsu segmentation is one of the most commonly applied methods for segmentation [15, 21, 22]. We used the green color channel because it gave best performance and it has been used in many of the previous studies. We developed a new two-step Otsu segmentation method as this gave better results than a simple Otsu segmentation. The two steps are as follows: First, we applied Otsu’s method to detect background (parts of the image not covered by RBC or WBC) and foreground (red blood cells and parasites). Then we inverted the intensity of the background. Second, we applied three-level Otsu [23] segmentation on the background-inverted image. We used the lowest threshold from the second step on the green channel to segment the parasites. On the segmented image we applied the morphological operations of filling with 8-connected background neighbors and of dilation with a disk structure with radius of 5 (same as used for pixel classification).

### Software packages

We used R (version 3.2.1) [24] and the package RandomForest (version 4.6-10) which is based on Breiman’s random forest algorithm [25, 26]. We used 100 trees for pixel classification. We used the EBImage package for Gaussian blur and feature calculations for stage classification [20]. We used a Random Forest with 500 trees for stage classification. We used Matlab r2014b for Otsu segmentation and for making the figures.

## Results

### Image segmentation

**Fig. 3:**
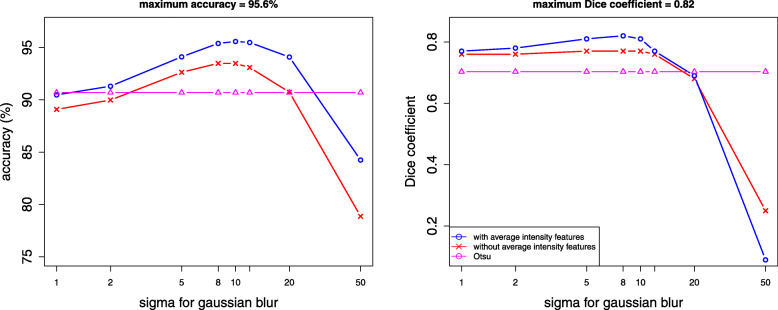
Performance of the pixel classifier with and without average intensity features for different standard deviations of Gaussian blur. Also shown is the performance of Otsu segmentation

We first set out to determine the optimal level of smoothing and whether the basic approach with 3 features for each pixel could be improved upon. Figure 3 shows how the cross-validated accuracy (left panel) and the Dice coefficient (right panel) vary with different standard deviations of the Gaussian blur. Figure 3 also shows the effect of inclusion of the average intensities of red blood cells and background. Results suggest that the pixel classifier performs better with average intensity features. We get an accuracy of 95.6*%* for a standard deviation of 10 pixels of the Gaussian blur.

The Dice coefficient is calculated over all pixels of all images unlike the accuracy which is calculated over a balanced data set only. The optimal value of the Dice coefficient is obtained for a standard deviation of 8 pixels both with and without average intensity features. The maximum value of the Dice coefficient is 0.76 without average intensities while with average intensities, we get a maximum of 0.82. Results show that inclusion of the average intensity features helps in correcting for the red blood cell (RBC) density and variability of staining and improves the accuracy as well as the Dice coefficient.

**Fig. 4:**
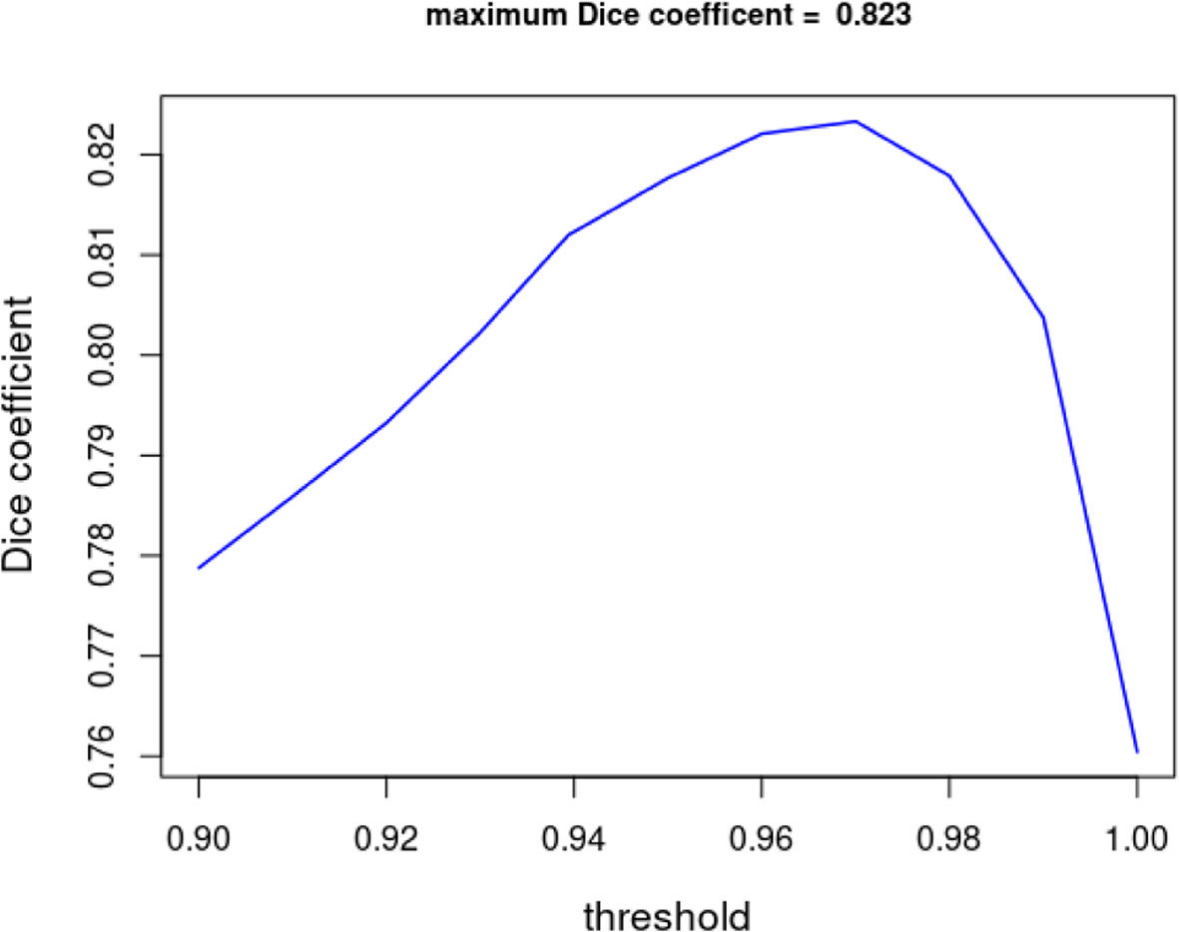
Dice coefficient as a function of probability threshold

Next, we explore how sensitive performance is for our choice of probability threshold of 0.98. Figure 4, which is made with a blurring factor of 8 pixels, shows that the optimal value of the Dice coefficient is 0.823 at threshold value of 0.973 which is close to the Dice coefficient of 0.821 obtained with the threshold value of 0.98. Performance is not strongly affected by the exact value of the probability threshold, provided one takes a threshold between 2 to 5 times the estimated fraction covered by parasites.

Figure 3 shows an accuracy of 90.7*%* with an average Dice coefficient of 0.70 using a two-step Otsu segmentation. The accuracy of the Otsu method is calculated from balanced data for comparison with the accuracy from pixel classification. The Dice coefficient of Otsu segmentation is about 13% lower than the Dice coefficient obtained with pixel classification. Thus pixel classification performs better than the Otsu method.

**Fig. 5:**
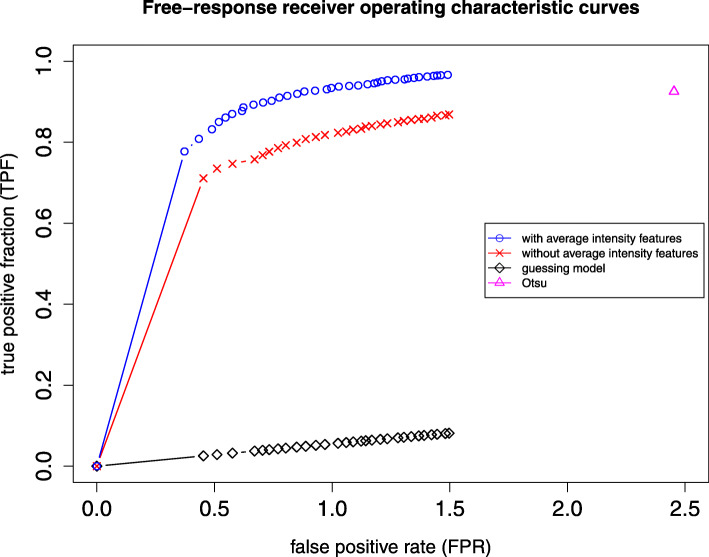
FROC curves for the detection and localization of parasites

Figure 5 shows FROC curves obtained with average intensity features, without average intensity features and by the Otsu method. With average intensity features, the difference of area between curves of the guessing model and the empirical data is 1.13 while without average intensity features, the difference is 0.97. These results also suggest that inclusion of the average intensity features improves the performance of pixel classification. Also shown is the performance of three-level Otsu segmentation in Fig. 5. This achieves a true positive fraction of 0.925 at the expense of a high false positive rate of 2.45. In contrast the pixel classifier with average intensity features achieves a true positive fraction of 0.925 for a false positive rate of 0.92, a two and a half fold reduction relative to Ostu segmentation.

### Life stage classification

By performing stage classification separately on each color channel, we find that the green channel gives slightly better performance than the red and blue channels. We get accuracies of 55%, 54% and 53% for the green, blue and red channels respectively. When we perform stage classification with all channels, we find an accuracy of 58.8*%* which is better than the performance with individual channels. Table 4 shows the confusion matrix obtained from the classifier. We see from Table 4 that life stages are mostly confused with their neighboring stages. For example, LR-ET is mostly confused with the R and MT stages. Similarly MT is mostly confused with LR-ET and LT. On combining, R with LR-ET, MT with LT and Esch with Lsch, we get an accuracy of 82.7*%*. If we exclude WBCs and debris from the stage classification, we are left with only parasite stages. Classification performance is about 60% when excluding WBCs and debris, which shows that these classes improve performance. Finally we performed stage classification with 11 important features. These features were area, perimeter, standard deviation, mean intensities and contrast features of the red, green and blue channels. We get an accuracy of 57% with these features which is only slightly lower than the accuracy with all features.

**Table 4:** Average confusion matrix with overall accuracy of 58.8*%*

	R	LR-ET	MT	LT	Esch	Lsch	Seg	WBC	Debris	Accuracy(%)
R	385	31	8	4	10	6	2	1	91	71.6
LR-ET	49	179	32	6	2	1	0	0	38	58.3
MT	8	51	188	52	8	0	1	0	24	56.6
LT	6	18	67	145	112	6	0	0	13	39.5
Esch	11	5	18	72	484	81	14	0	29	67.8
Lsch	8	2	8	6	212	165	32	0	13	37.0
Seg	3	1	6	2	50	39	95	0	11	45.9
WBC	1	0	0	0	1	1	0	42	5	84.0
Debris	102	30	22	14	37	5	4	7	460	67.6

## Discussion

We constructed a dataset with almost 3000 segmented and labeled Plasmodium falciparum parasites. We publicly share this data set as a resource for future studies ([27]).

This study proposes parasite segmentation using a random forest classifier for pixel classification. We evaluated our proposed segmentation approach with three metrics: cross-validated accuracy, Dice coefficient and FROC. All metrics suggest that pixel classification is better than the Otsu method and that inclusion of average intensity features improves performance of the pixel classifier. The proposed method also generalizes to unseen images of Plasmodium falciparum obtained from the website of the Centers for Disease Control and Prevention (CDC). Results from the CDC images are presented in the Appendix.

The free-response receiver operating characteristic (FROC) is a generalization of ROC analysis to the case where the number of false positives can be larger than 1. It is commonly used for the evaluation of diagnostic systems. None of the previous studies in Table 2 used FROC analysis for evaluation of their results. FROC analysis quantifies the trade-off between true and false positives. True positives are those segmented images patches that have a minimum overlap with a ground truth patch, where the minimum overlap is defined as a Dice coefficient of 0.2. Our results show that FROC analysis can be used for malaria parasite detection to illustrate the trade-off between true and false positives. FROC analysis shows that pixel classification performs considerably better than Otsu segmentation as the number of false positives for pixel classification is less than half the number for Otsu segmentation for the same true positive fraction.

Most of the previous studies on malaria parasite detection were performed by thresholding or by morphological methods as detailed in Table 2 and [15]. Some recent studies [6, 7] applied deep learning and neural network methods to classify red blood cells into healthy or infected ones. Rajaraman et al. [7] uses a level-set based method and multi-scale Laplacian of Gaussian (LoG) filter to segment red blood cells as opposed to direct detection of malaria parasites with pixel classification in our study. Their study shows that a pre-trained ResNet-50 outperforms other deep learning methods and that the last layer of the neural network does not always give optimal features to classify cells. The accuracy of their method is 98.6*%* while current study reports an accuracy of 95.6*%* without the extra step of segmentation of red blood cells. Sorgedrager [6] applies pixel classification with a convolutional neural network to classify parasite or non-parasite pixels and it reports an accuracy of 97.3*%* but the study is limited to only ten images.

Most of the previous studies report life stage classification with ring, trophozoite and schizont [28–30], but the current study reports it with sub-stages. The accuracy of stage classification is relatively low because sub-stages are close to each other in appearance, which can be observed from the confusion matrix (Table 4). We find a classification accuracy between 55 and 60%. To put this accuracy in perspective we had a second expert classify a balanced subset of 115 parasites. The agreement between the two experts was 38%. The agreement between the classifier and the first expert was 51% while agreement between the classifier and the second expert was 29%. The agreement between the classifier and the first expert is naturally higher because the classifier was trained on data from the first expert. The agreement is lower than the reported accuracy of 58.8*%* (on all of the data) because the subset was apparently more difficult to classify. Importantly, the results of the second expert show that the task is difficult and that the average performance of our proposed life stage classification method (40% = average agreement with first and second expert) is comparable to the agreement between experts. Ruberto et al. [30] also reports disagreement among experts on life stage classification of parasites. By combining life stages in only three classes, ring, trophozoite and schizont, our classification accuracy is 82.7*%* which is comparable to the accuracy of 89% and 84.87*%* as reported by Boray et al. [31] and Diaz et al. [28]. Current study reports results of stage classification on 2911 parasites as whereas [31] reports 669 and [28] 243 parasites. Rosado et al. [4] reports accuracies of 73.9*%* and 94.8*%* for classification of trophozoites and gametocytes with 585 and 58 parasites respectively. Previous studies were performed with relatively small datasets for stage classification while the current study explores performance with a relatively large dataset. Lastly, previous studies report slightly better results on stage classification possibly because they were performed with images of in-vitro cultured parasites, that have less variation and fewer artifacts than field study parasites.

In future studies, it would be interesting to explore other methods such as deep learning for both pixel segmentation and stage classification. It would also be interesting to explore segmentation and classification of different species of the malaria parasite. Based upon the results and algorithms developed in the current study, it might be feasible to develop a mobile application for the detection and stage classification of malaria parasites from Giemsa stained images. Such an application would be helpful in areas where there is a shortage of resources for early detection of malaria.

## Conclusion

In summary, we find that the inclusion of the average intensities as features in the pixel classification improves segmentation. Our method outperforms traditional segmentation methods. FROC analysis is used for the first time in the study of malaria image analysis for the object-level evaluation of segmentation. Lastly, we publicly share our data set of original images, ground-truth segmentation and life stage classification as a resource for future studies.

## Appendix: CDC data set

The Centers for Disease Control and Prevention (CDC) report exemplary images of Plasmodium falciparum on their website [33]. We used these CDC images as a validation data set to evaluate our pixel classification approach. These images were collected from different sources and were not used for training so can function as an independent validation. We downloaded 13 images of thin blood films from the CDC website. Each image was 300× 300 pixels, 8-bit RGB and JPEG-encoded. We manually created a ground truth segmentation for these images with an identical procedure as for the Lemieux data set. We found a total 39 malaria parasites (infected red blood cells). The parasites were classified in three life stages (ring, trophozoite or schizont) by the CDC. All parasites in an image were in the same life stage. There were 5 images with rings, 5 images with trophozoites and 3 images with schizonts.

An issue with the images from the CDC is that they have different and unknown magnifications of the microscope used to image the thin blood film slides. In order to adjust for this, we used the size of red blood cells as yardstick. We measured the diameter of 50 red blood cells in our training images and found the average diameter to be 124±5 pixels. We similarly measured the diameter of four red blood cells in each of the CDC images and found it to vary from 40.3 to 69.5 pixels. We used the ratio of red blood cell diameters to scale the standard deviation of the Gaussian blur. We then blurred the CDC images with this scaled standard deviation and used the trained random forest classifier to segment them. We trained our random forest classifier on data from all patients (without cross validation). As we did in training, we removed all segmented objects smaller than 100 pixels scaled by the same red blood cell diameter based scaling factor. The 100 pixels minimum size stems from the smallest parasite (100 pixels) in the training images (See methods: Image features and pixel sampling).

In supplementary data, we show the results of segmenting the CDC images as overlapping images of segmentation and ground truth. Segmented pixels are colored green, ground truth pixels are colored red while the overlapping ones are colored yellow. The title of each image gives the name of the image file (from the CDC web site) and number of detected and infected red blood cells. We chose to report the number of detected and infected red blood cells as opposed to the number of detected and present parasites as many red blood cells were infected with multiple parasites. Multiple infection of red blood cells is considered to be an artifact of in-vitro infection and is rare in-vivo. As our pixel classifier segmentation is trained on in-vivo-like data, we did not consider the multiple infection artifact. Also reported in the title of each image is Dice coefficient. The average Dice coefficient over all images is 0.66 (median value is 0.72) which is lower than the cross-validated mean value of 0.82 found for the training images. Despite this lower Dice coefficient it is clear from inspection of the CDC images that our segmentation method generalizes well to unseen data.

## Supplementary information

**Additional file 1** CDC data set.

## Data Availability

The data is available in the Malaria-Detection-2019 repository [27].

## Abbreviations

CDC: Centers for Disease Control and Prevention
FPR: False Positive Rate
FROC: Free-response Receiver Operating Characteristic
RBC: Red Blood Cell
ROC: Receiver Operating Characteristic
TPF: True Positive Fraction
WBC: White Blood Cell
R: Ring (parasite life stage)
LR-ET: Late ring or early trophozoite (parasite life stage)
MT: Mid trophozoite (parasite life stage)
Esch: Early schizont (parasite life stage)
Lsch: Late schizont (parasite life stage)
Seg: Segmenter (parasite life stage)
