# Supplementary material for: Detection and stage classification of Plasmodium falciparum from images of Giemsa stained thin blood films using random forest classifiers
Source: Diagn Pathol. 2020 Oct 23;15:130. doi: 10.1186/s13000-020-01040-9 (PMC7585298; doi:10.1186/s13000-020-01040-9)

Pf_rings_thinA

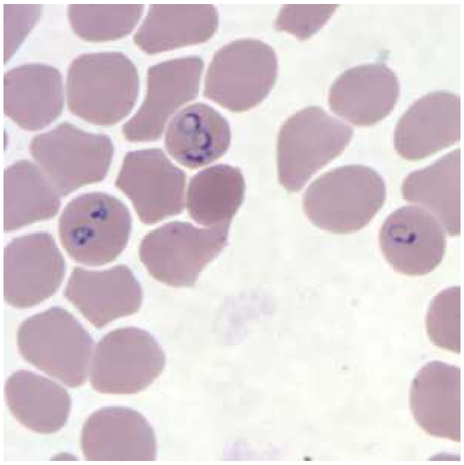

dice = 0.53 parasites = 4/3

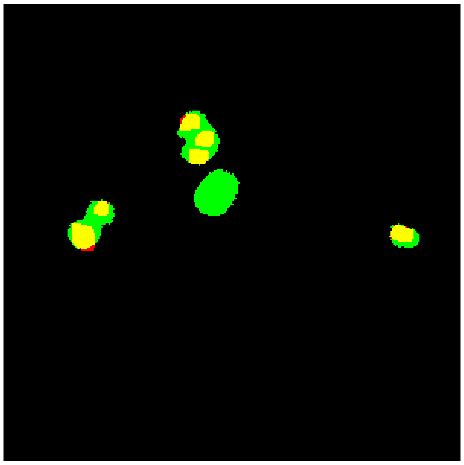

Pf_rings_thinB

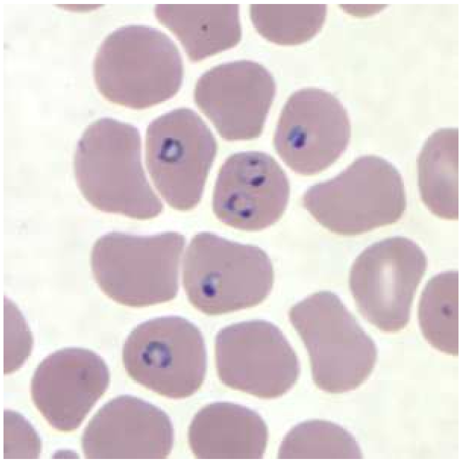

dice = 0.79 parasites = 7/4

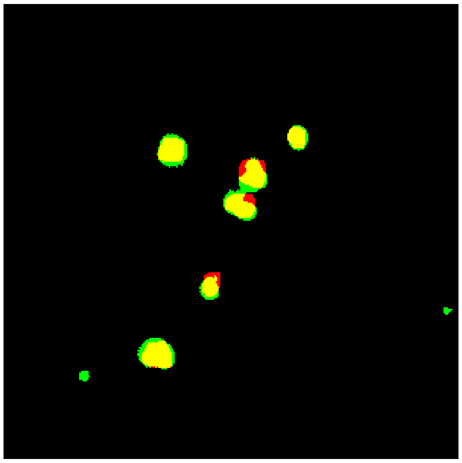

Pf_rings_thinC

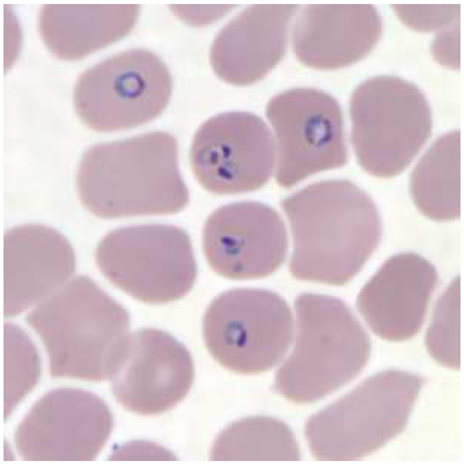

dice = 0.71 parasites = 5/4

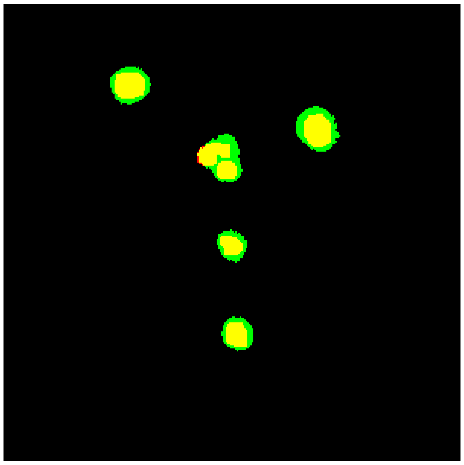

Pf_rings_thinD

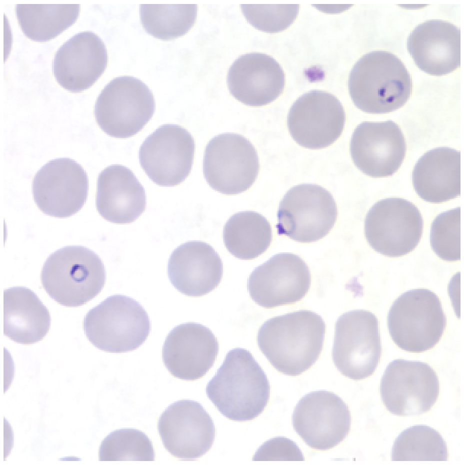

dice = 0.61 parasites = 2/3

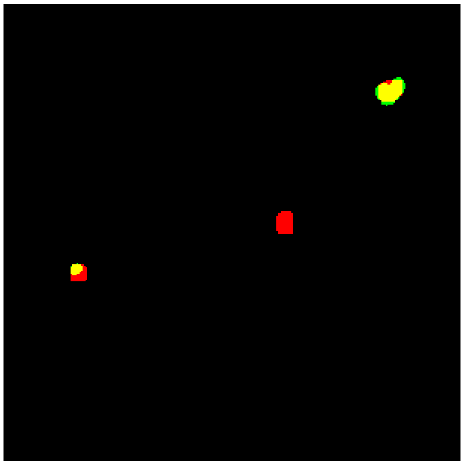

Pf_rings_thinE

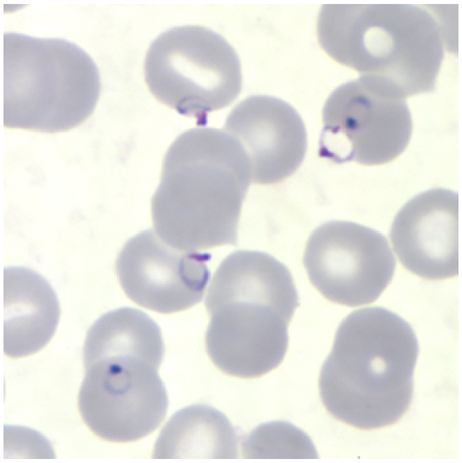

dice = 0.36 parasites = 8/4

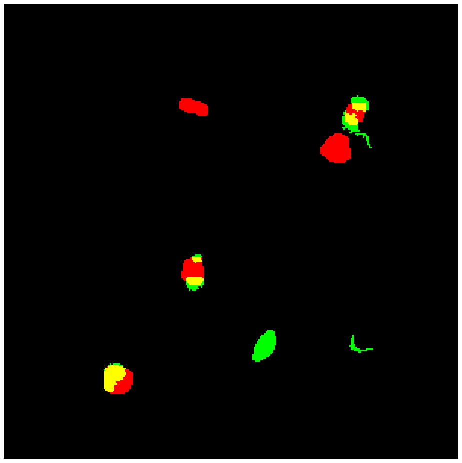

Pf_schizont_thinA

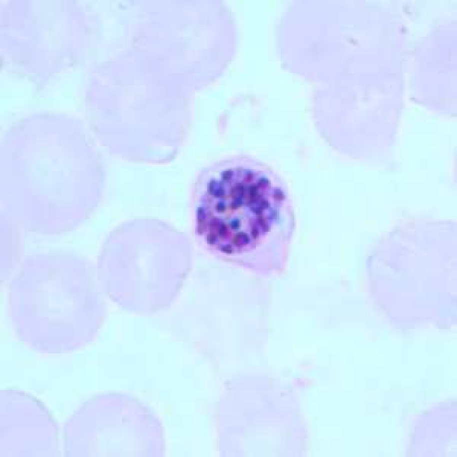

dice = 0.96 parasites = 1/1

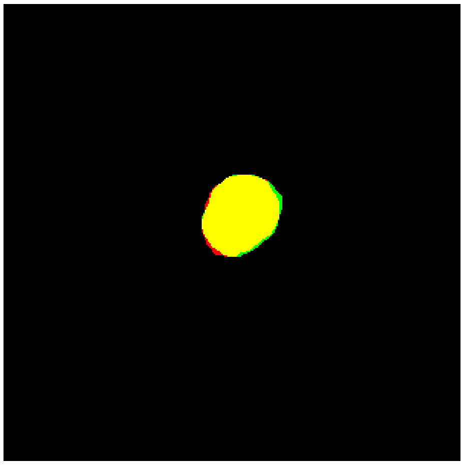

Pf_schizont_thinB

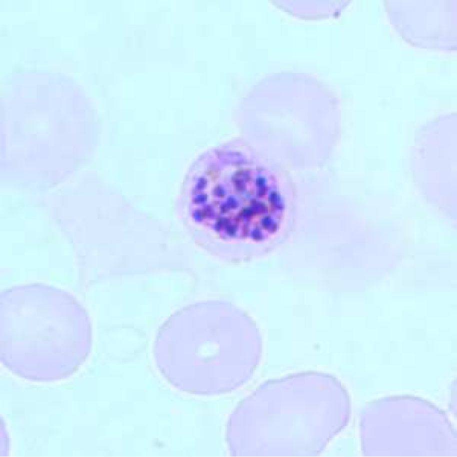

dice = 0.88 parasites = 1/1

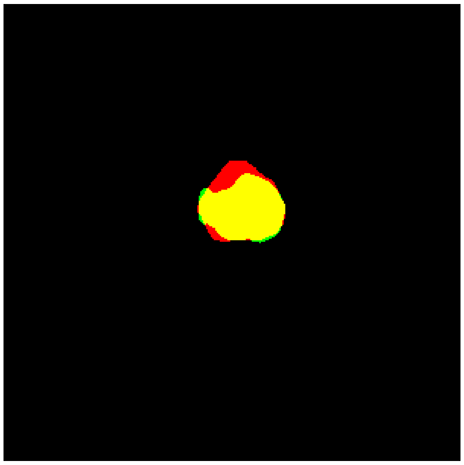

Pf_schizont_thinC

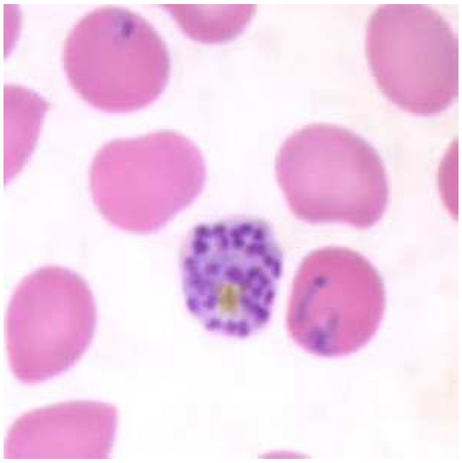

dice = 0.27 parasites = 2/1

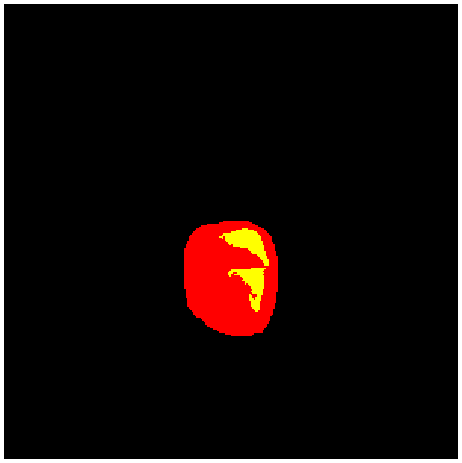

Pf_troph_thinA

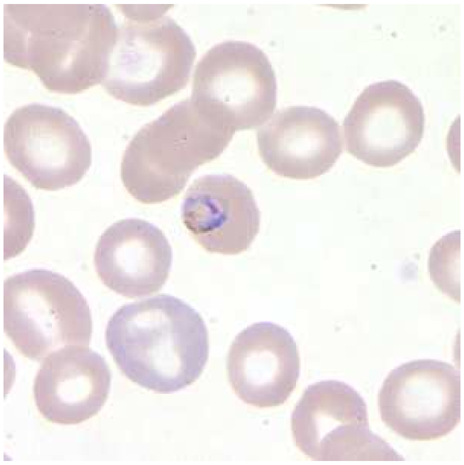

dice = 0.57 parasites = 1/1

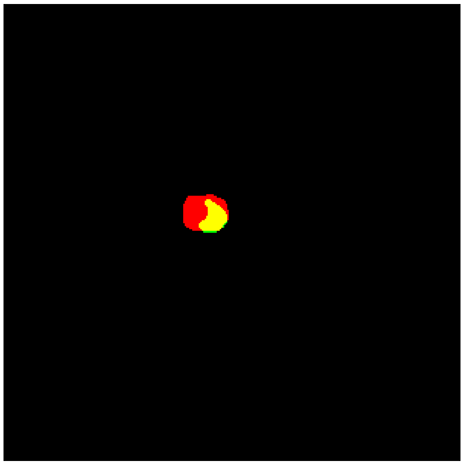

Pf_troph_thinB

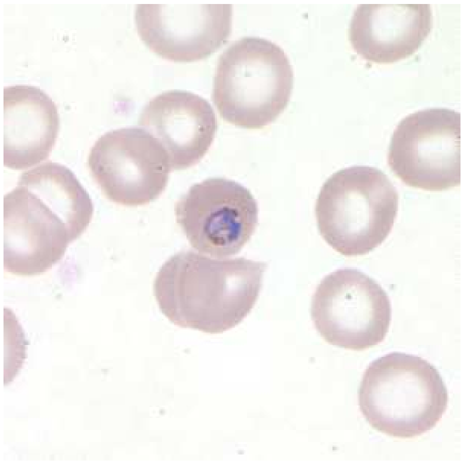

dice = 0.76 parasites = 1/1

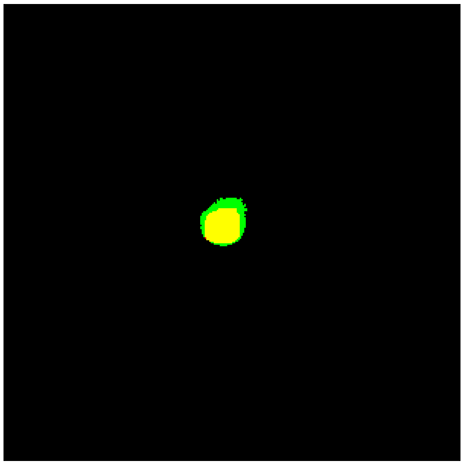

Pf_troph_thinC

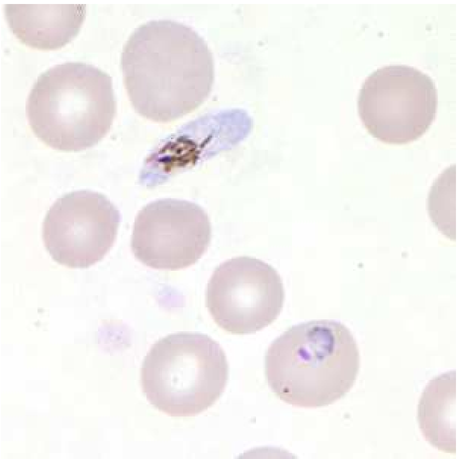

dice = 0.54 parasites = 1/2

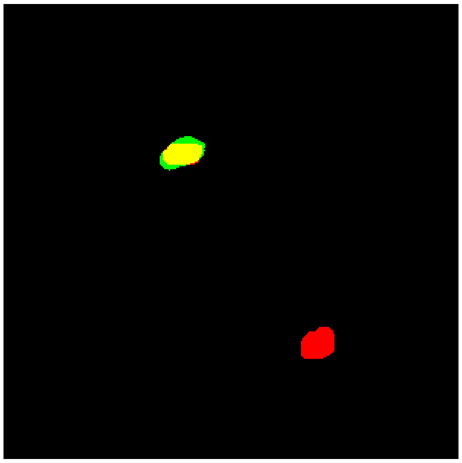

Pf_troph_thinD

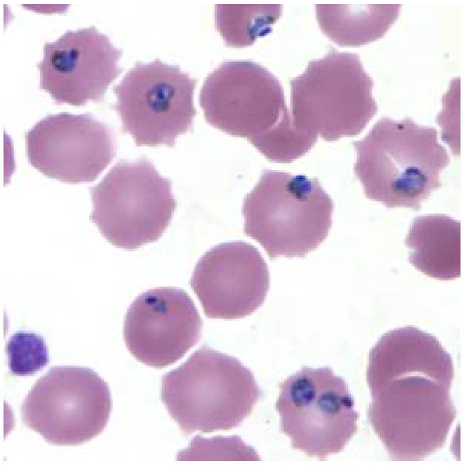

dice = 0.84 parasites = 9/8

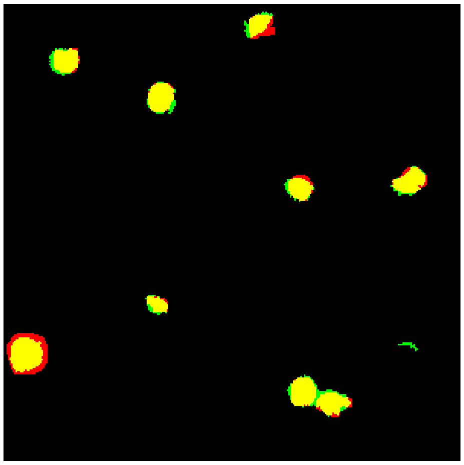

Pf_troph_thinE

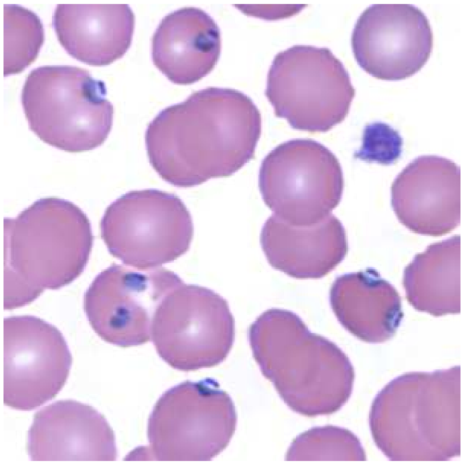

dice = 0.76 parasites = 9/6

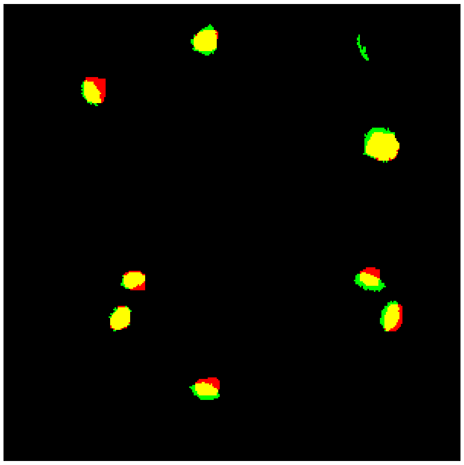

Supplement: Supplementary file 1 — Additional file 1 CDC data set. [file 13000_2020_1040_MOESM1_ESM.pdf]
